# Interpersonal Interactions and Affect in Daily Life Among Midlife and Older Adults

**DOI:** 10.1093/geroni/igab046.2842

**Published:** 2021-12-17

**Authors:** Robert Stawski, Dakota Witzel, Madeline Nichols, Susan Charles

**Affiliations:** 1 Oregon State University, Corvallis, Oregon, United States; 2 University of California, Irvine, Irvine, California, United States

## Abstract

Theories of age and emotional wellbeing posit that older age is associated with better affective well-being through avoidance or minimization of distressing experiences and prioritizing positive experiences and emotions. To test these theories, researchers have examined change in affect (i.e., reactivity) associated with negative interpersonal experiences in daily diary studies, given the compromising effects these interpersonal stressors exert on daily affect. In contrast, age differences in the potential affect-enhancing effects of positive interpersonal experiences have been comparatively neglected. Using the second wave of the National Study of Daily Experiences, we evaluated age differences in the frequency of daily negative and positive interpersonal interactions, as well as the affective responses to these interpersonal interactions. Positive and negative affect, as well as negative and positive interpersonal interactions were assessed on eight consecutive evenings. Analyses included 818 participants (Mage=53.3, SD=11.8, Range=34-83; 60% female) who experienced both negative and positive interpersonal interactions during the 8-day protocol. Preliminary results revealed increased frequency of negative interpersonal interactions and decreased frequency of positive interpersonal interactions with age (ps<.01). Further, negative interpersonal interactions were associated with increases in negative affect and decreases in positive affect (ps<.01), while positive interpersonal interactions were associated only with increased positive affect (p<.01). Finally, modest evidence of age-related reductions in the affective impact of negative, but not positive, interpersonal interactions emerged (p=.03). Discussion will focus on how studies of interpersonal interactions in daily life can inform theories of aging and promote emotional wellbeing throughout adulthood and later life.

